# Analysis of related factors between the occurrence of secondary epidermoid cyst of penis and circumcision

**DOI:** 10.1038/s41598-022-16876-y

**Published:** 2022-08-09

**Authors:** JiangFan Yu, Rui Tang, Ke Ding

**Affiliations:** 1 Department of Dermatology, Second Xiangya Hospital, Central South University, Changsha, Hunan China; 2 Department of Rheumatology and Immunology, Second Xiangya Hospital, Central South University, Changsha, Hunan China; 3 Department of Urology, Xiangya Hospital, Central South University, Changsha, Hunan China

**Keywords:** Risk factors, Infection, Comorbidities, Trauma

## Abstract

Secondary epidermoid cyst of the penis is a very rare epidermoid cyst that occurs in the penis. The purpose of this study was to investigate the relationship between the occurrence of secondary epidermoid cyst of penis and circumcision-related factors, and to provide possible reasonable and effective suggestions for circumcision. The data of all patients who visited the clinic for epidermoid cysts of the penis from September 2000 to September 2021 in Xiangya Hospital were collected. A retrospective study was carried out on whether the patients had been circumcised and the surgical method, anesthesia method, cyst location, surgical age, postoperative wound infection, whether they were phimosis patients, and the level of the surgeon. Among the 24 patients followed up, 95.8% had a history of circumcision, and only 4.2% had no history of circumcision, and the more traumatic surgical methods developed secondary epidermoid cyst of the penis after surgery the higher the probability. Injecting anesthesia at the base of the penis increases the chances of developing a secondary epidermoid cyst of the penis. Postoperative secondary epidermoid cyst of the penis were mainly located in the anterior segment and posterior segment, and the anterior segment had a higher proportion, followed by the posterior segment. Secondary epidermoid cyst of the penis occur mainly in adults. Postoperative wound infection accelerates the appearance of secondary epidermoid cyst of the penis. Patients with phimosis have an increased probability of developing secondary epidermoid cysts of the penis after surgery. The incidence of secondary epidermoid cysts and postoperative infection after manual circumcision by the attending physician was higher than that of the chief physician. Circumcision, injection of anesthesia at the base of the penis, ligation of the penis, and postoperative wound infection may be the etiologies and triggers of secondary epidermoid cysts of the penis. Adults and phimosis patients may be high-risk groups. Lower-level surgeons may increase the odds of postoperative secondary epidermoid cysts of the penis, and it is recommended that surgery be performed by a clinically-experienced, higher-level surgeon. The indications for circumcision should be strictly evaluated and the operation should be performed as soon as possible, and the less invasive surgical method and anesthesia method should be selected. Reduce irrelevant operations during surgery and avoid wound infection after surgery.

## Introduction

Epidermoid cysts are one of the common benign lesions in dermatology, accounting for about 90% of resectable cysts^1,2^. The affected population is mainly adults^3,4^, and it can occur in any part, mainly in the head, neck and trunk^5,6^. Epidermoid cysts rarely worsen^7–9^, but rare malignancies may develop over time^3,10^. Epidermoid cysts of the penis are one of the types of epidermoid cysts, but they are very rare and usually painless^11^. The clinical manifestations are mainly the progressive enlargement of the cyst, and inflammatory pain may be manifested after infection or rupture of the cyst wall^4^. Treatment is based on complete surgical excision. At present, the etiology of penile epidermoid cysts is still controversial^12^, and there are reports in the literature that it may be caused by residual congenital material, or damage to the hair follicle structure due to trauma, infection, and other reasons^13–15^. The occurrence of secondary epidermoid cysts of the penis after circumcision has been noted, and so far there has never been any literature on the specific relationship between the occurrence of secondary epidermoid cysts of the penis and circumcision. The purpose of this study was to specifically analyze the relationship between secondary epidermoid cysts of the penis and circumcision-related factors, and to provide possible reasonable and effective suggestions for circumcision.

## Methods

The data of all patients with epidermoid cysts of the penis from September 2000 to September 2021 in Xiangya Hospital were collected. Whether the patient has been circumcised and the surgical method, anesthesia method, cyst location, surgical age, postoperative wound infection, whether it is a phimosis patient, and the level of the surgeon were investigated. In this study, a total of 27 patients with penile epidermoid cysts were identified. Among them, 2 patients could not be contacted due to wrong phone numbers, and 1 patient refused to communicate and did not complete the follow-up, so only 24 patients were finally investigated. Among them, 23 patients had a history of circumcision, and 1 patient had no history of circumcision.

### Ethical approval

This study has been reviewed by the Ethics Committee of Xiangya Hospital of Central South University, and the ethics number is: 202,111,212. Due to the spread of COVID-19 during the follow-up period, no written informed consent was signed, but the patient was informed in detail by telephone and verbal consent was obtained from the patient himself or his legal guardian. As the follow-up took place during a special period of covid-19 transmission, verbal consent was also approved by the Ethics Committee of Xiangya Hospital of Central South University. All data in this study were obtained from patient dictation. This study was conducted in strict accordance with the Declaration of Helsinki.

### Informed consent

This study uses coding to hide all identifiable information of patients, and will follow the principles of scientific research risk control, privacy protection, and special protection. This research promises to comply with the requirements of scientific research management standards, and the research content will not cause harm and risk to the research subjects. The research subjects will be based on the principle of voluntariness, the right to know is guaranteed, and the rights and privacy of the research subjects will be protected to the greatest extent possible.

## Results and discussion

The etiology of penile epidermoid cyst has not been fully clarified. It has been reported in the literature that after neonatal circumcision, secondary epidermoid cysts of the penis may occur as a complication^16^, and the specific mechanism has not been studied.According to the etiology, epidermoid cysts of the penis are roughly divided into congenital and secondary epidermoid cysts of the penis^17^. Congenital epidermoid cysts of the penis may result from abnormal closure of the embryonic median raphe^18,19^. According to relevant literature reports, the occurrence of secondary epidermoid cysts of the penis may be due to trauma, surgery, and inflammation^20–22^, but no further research and analysis were conducted, which is also the significance of this study (Table 1). In this study, we found that regardless of circumcision or injection of anesthesia at the root of the penis, the incidence of postoperative secondary epidermoid cysts of the penis will increase, and the more traumatic surgical methods, the incidence of secondary epidermoid cysts of the penis (Tables 2 and 3). This may be related to the implantation of epidermal tissue into the dermis, occlusion of hair follicles, and obstruction of exocrine ducts after surgery and invasive procedures with injection of anesthesia at the base of the penis^23^. Patients undergoing three different surgical procedures were observed and found to have the highest incidence of secondary epidermoid cysts after manual circumcision (Table 3). The main reason may be that manual circumcision involves more operative steps than ligation and stapler circumcision. The ligation circumcision and stapler circumcision can be sutured at the same time as the overly long foreskin is removed. The time is short, the speed is fast, the effect is ideal, and there is no need for repeated operations. Manual circumcision requires a rubber band to ligate the base of the penis before the operation to reduce bleeding during the operation. After that, the overly long foreskin needs to be completely excised, and then the wound is sutured intermittently. In this process, the skin of the penis needs to be pulled and fixed with toothed forceps repeatedly. The presence of these invasive procedures described above greatly increases the risk of epidermal tissue implantation into the dermis, postoperative hair follicle occlusion, exocrine duct occlusion, and postoperative infection. This may be the main reason for the higher incidence of secondary epidermoid cysts after manual circumcision. In this study, all patients undergoing manual circumcision had the base of the penis bandaged with a rubber band before surgery to reduce bleeding, a procedure that may result in damage to the skin of the penis, which may promote secondary. The occurrence of penile epidermoid cysts, but the specific pathogenesis needs further study. From the location of the secondary epidermoid cysts of the penis, we found that when the secondary epidermoid cysts of the penis occurred in the posterior segment, the anesthesia was injected into the root of the penis, which may be related to the epidermal injection debris during the injection of anesthesia at the root of the penis^24^ (Table 4). Therefore, whether we can consider using other anesthesia methods to replace penile root injection anesthesia, which not only relieves the pain of anesthesia, but also reduces the occurrence of secondary epidermoid cysts of the penis after surgery.Relevant literature reports that the incidence of secondary epidermoid cysts of the penis is mainly adults^3,4^. Among the 23 patients with a history of circumcision in our study, 19 were adults and only 4 were minors (Tables 1 and 5). Is there a higher risk of secondary epidermoid cysts of the penis after adult circumcision?To avoid postoperative secondary epidermoid cysts of the penis, should surgery be recommended for male patients with indications for circumcision in adolescence? Of course, this requires more data to illustrate. Post-operative wound infection is a common complication after circumcision^25^, and post-circumcision wound infection may lead to occlusion of hair follicles or obstruction of exocrine ducts^22^, thereby increasing the occurrence of secondary epidermoid cysts of the penis. In this study, we did find that in patients with postoperative infection, the appearance of secondary epidermoid cysts of the penis was earlier (Tables 6 and 7), so the prevention of infection after circumcision is very important.Phimosis is one of the indications for circumcision, and it has been reported that secondary epidermoid cysts of the penis occurred in patients after phimosis^23^. In this study, we found that patients with phimosis had a higher rate of secondary epidermoid cysts of the penis (Table 8), probably due to the need for more invasive procedures during surgery in patients with phimosis. Therefore, it is necessary to explain the postoperative complications to the phimosis patients in detail before the operation, and avoid irrelevant invasive operations as much as possible during the operation, so as to avoid the probability of postoperative secondary epidermoid cysts of the penis.We found a higher incidence of secondary epidermoid cysts and postoperative infections after manual circumcision in the attending physician than in the chief physician (Table 9). This may be related to the lack of clinical experience of the attending physician, and there are many steps in manual circumcision. Low-level surgeons added ineffective manipulations in the process, further damaging the skin of the penis. It is also possible that with the increase of operation steps and the increase of operation time, the probability of infection is increased, and postoperative infection is more likely to occur, thereby inducing secondary epidermoid cysts of the penis. The diagnosis of secondary epidermoid cyst of the penis is easily confused with lipomas, fatty cysts, dermoid cysts, boils, and carbuncles^26^. A diagnosis must be made before treatment in order to decide on a treatment plan. Diagnosis mainly depends on the following points: (1) Medical history: whether there is a history of trauma and inflammation, especially whether there is a history of circumcision (2) Physical examination: a skin mass with a clear boundary can be seen on inspection, and a painless palpable mass can be palpated by palpation. Non-volatile compressible substances (3) Auxiliary examination: round or oval hypoechoic mass under color Doppler ultrasound (4) Pathological examination: keratin filling, fibrous tissue coverage^27^, secretory cell formation under immunohistochemistry Factors 1 and 10^28^, the most important of which is to carry out pathological examination. The main treatment is surgery, especially after it is known that it may turn into a malignant direction, such as squamous cell carcinoma and basal cell carcinoma^29–31^. Most of the local anesthesia surgery can be successfully resected, a small part requires general anesthesia, in order to avoid recurrence, the cyst wall must be completely removed. All surgically removed secondary epidermoid cysts of the penis should be confirmed histopathologically ^32^. Since secondary epidermoid cysts are very rare, this study was limited by the limited number of cases collected. However, we found some association and regularity between secondary epidermoid cysts and circumcision-related factors in a limited number of cases, which is a very meaningful study for surgeons. This will help surgeons to make more reasonable choices in circumcision operation methods, anesthesia methods, operation age, and selection of surgeons, and make clearer recommendations on the prevention of postoperative infection.

**Table 1:** Basic information of patients.

Case	Age	BMI	Whether there is a history of circumcision	Age of surgery	Postoperative appearance time (months)	Location	Symptom	Treatment department	Treatment
1	27	28.1	Yes	21	6	Dorsal aspect (8 and 12 o’clock)	Painless nodules (progressive enlargement)	Dermatology	Surgery
2	48	26.5	Yes	34	4	Right side of the corona	Painless nodules (progressive enlargement)	Urology	Surgery
3	39	26.0	Yes	37	12	Right side of the corona	Painless nodules (progressive enlargement)	Urology	Surgery
4	43	19.9	Yes	24	5	Dorsal aspect (12 o’clock)	Painless nodules (progressive enlargement)	Urology	Surgery
5	20	22.2	Yes	19	15	Left side of the corona	Painless nodules (progressive enlargement)	Urology	Surgery
6	49	26.9	Yes	45	10	Dorsal aspect (7 and 10 o’clock)	Painless nodules (progressive enlargement)	Urology	Surgery
7	37	18.1	Yes	22	6	Right side of the corona	Painless nodules (progressive enlargement)	Urology	Surgery
8	23	25.8	Yes	17	8	Dorsal aspect (9 and 2 o’clock)	Painless nodules (progressive enlargement)	dermatology	Surgery
9	28	26.3	Yes	23	9	Entire circumference of the corona	Painless nodules (progressive enlargement)	Urology	Surgery
10	34	24.8	Yes	20	7	Right side of the penis at the line where the foreskin base had been removed	Painless nodules (progressive enlargement)	Urology	Surgery
11	36	20.7	Yes	19	7	Left side of the corona	Painless nodules (progressive enlargement)	Urology	Surgery
12	41	29.1	Yes	28	6	Ventral aspect of the corona(6 o’clock)	Painless nodules (progressive enlargement)	Urology	Surgery
13	19	19.7	Yes	18	11	Dorsal aspect (7 and 11 o’clock)	Painless nodules (progressive enlargement)	Urology	Surgery
14	22	17.4	Yes	16	8	Ventral aspect of the corona	Painless nodules (progressive enlargement)	Urology	Surgery
15	31	27.7	NO	–		Dorsal aspect (8 and 11 o’clock)	Painful nodules (progressive enlargement)	Urology	Surgery
16	46	20.1	Yes	35	7	Right side of the corona	Painless nodules (progressive enlargement)	Urology	Surgery
17	27	19.7	Yes	24	11	Right side of the corona	Painless nodules (progressive enlargement)	Urology	Surgery
18	50	16.1	Yes	43	8	Entire circumference of the corona	Painless nodules (progressive enlargement)	dermatology	Surgery
19	34	19.6	Yes	32	9	Ventral aspect of the corona (4 and 7 o’clock)	Painless nodules (progressive enlargement)	Urology	Surgery
20	14	26.7	Yes	13	13	Dorsal aspect (11 o’clock)	Painless nodules (progressive enlargement)	Urology	Surgery
21	43	25.9	Yes	27	9	Left side of the corona	Painless nodules (progressive enlargement)	Urology	Surgery
22	31	24.6	Yes	25	9	Left side of the penis at the line where the foreskin base had been removed	Painless nodules (progressive enlargement)	Urology	Surgery
23	25	25.5	Yes	17	7	Ventral aspect of the corona (4and6 o’clock)	Painless nodules (progressive enlargement)	Urology	Surgery
24	17	24.7	Yes	13	10	Right side of the corona	Painless nodules (progressive enlargement)	Urology	Surgery

**Table 2:** Incidence of secondary epidermoid cysts of the penis after surgery and different surgical modalities.

	Surgical patients			Non-surgical patients
Surgical procedure	Manual circumcision	Ligature circumcision	Stapler circumcision	–
Quantity (percentage)	15 (62.5%)	6 (25.0%)	2 (8.3%)	1 (4.2%)
Total		23 (95.8%)		1 (4.2%)

The proportion of patients with secondary epidermoid cysts of the penis after circumcision was significantly higher than that in non-circumcised patients. Among the three methods of circumcision, the proportion of secondary epidermoid cysts of the penis after manual circumcision was the highest, which was significantly higher than that of ligation and stapler circumcision.

**Table 3:** Different anesthesia methods have different proportions of secondary epidermoid cysts of the penis after operation.

	Penis root injection anesthesia	Lidocaine ointment anesthesia				
Quantity (percentage)	15 (65.2%)	8 (34.8%)				

	Manual circumcision		Ligature circumcision		Stapler circumcision	
Anesthesia	Penis root injection anesthesia	Lidocaine ointment anesthesia	Penis root injection anesthesia	Lidocaine ointment anesthesia	Penis root injection anesthesia	Lidocaine ointment anesthesia
Quantity (percentage)	10 (43.5%)	5 (21.8%)	4 (17.4%)	2 (8.7%)	1 (4.3%)	1 (4.3%)

Among the 23 surgical patients, regardless of the total proportion or specific surgical methods, the proportion of secondary epidermoid cysts after penile root injection anesthesia was higher than that of lidocaine cream anesthesia.

**Table 4:** Anesthesia methods and the location of secondary epidermoid cysts of the penis.

	Front section						Middle section						Back section					
Operation	A		B		C		A		B		C		A		B		C	
Anesthesia	D	E	D	E	D	E	D	E	D	E	D	E	D	E	D	E	D	E
Quantity	4	4	2	2	0	0	1	2	0	0	0	0	4	0	2	0	2	0

A: Manual circumcision, B: Ligature circumcision, C: Stapler circumcision, D: Penis root injection anesthesia, E: Lidocaine ointment anesthesia.

The penis is divided into three segments, and statistics show that the anterior segment (near the glans penis), the middle segment, and the posterior segment (near the abdomen) account for 52.2%, 13.0%, and 34.8%, respectively. When the secondary epidermoid cysts of the penis occurs in the posterior segment, the anesthesia method is injection anesthesia at the base of the penis.

**Table 5:** The age at surgery and the occurrence of secondary epidermoid cysts of the penis.

	Manual circumcision		Ligature circumcision		Stapler circumcision	
Age of surgery	< 18	≥ 18	< 18	≥ 18	< 18	≥ 18
Quantity (percent)	2 (8.7%)	13 (56.5%)	1 (4.3%)	5 (21.7%)	1 (4.3%)	1 (4.3%)

Among the people who developed secondary epidermoid cysts of the penis after surgery, whether it was the total proportion or the proportion after different surgical procedures, the proportion of adult patients was the highest.

**Table 6:** The relationship between postoperative wound infection and secondary penile epidermoid cyst.

	Postoperative infection	No infection occurred after surgery
Quantity (percentage)	6 (26.1%)	17 (73.9%)

**Table 7:** The appearance time of postoperative wound infection and secondary epidermoid cysts of the penis.

	Manual circumcision		Ligature circumcision		Stapler circumcision	
Postoperative infection	Yes	No	Yes	No	Yes	No
Quantity	3	12	2	4	1	1
Mean time to appearance after surgery (months)	5.3	7.6	8.5	11.3	12	15

Among the 23 surgical patients, 26.1% had postoperative infection, and 73.9% had non-surgical infection. Statistical analysis of postoperative infection was performed according to the surgical procedure. Whether the same or different surgical procedures were compared, the presence of postoperative wound infection accelerated the appearance of secondary epidermoid cysts of the penis.

**Table 8:** Phimosis and secondary epidermoid cyst of the penis.

	Manual circumcision		Ligature circumcision		Stapler circumcision	
Phimosis	Yes	no	Yes	no	Yes	no
Quantity	8	7	4	2	1	1

Statistical analysis found that the presence of phimosis increased the probability of secondary epidermoid cysts of the penis regardless of the total proportion or the comparison of different surgical procedures.

**Table 9:** Surgeons and the occurrence of secondary epidermoid cysts of the penis.

	Attending physician						Chief physician					
Anesthesia	D			E			D			E		
Surgical methods	A	B	C	A	B	C	A	B	C	A	B	C
Number of secondary penile epidermoid cysts (percentage)	7 (30.4%)	2 (8.7%)	0	4 (17.4%)	1 (4.3%)	1 (4.3%)	3 (13.0%)	2 (8.7%)	1 (4.3%)	1 (4.3%)	1 (4.3%)	0
Number of postoperative infections (percentage)	3 (13.0%)	0	0	2 (8.7%)	0	0	1 (4.3%)	0	0	0	0	0

A: Manual circumcision, B: Ligature circumcision, C: Stapler circumcision, D: Penis root injection anesthesia, E: Lidocaine ointment anesthesia.

Statistics show that the incidence of secondary penile epidermoid cysts in the chief physician is lower than that in the attending physician, especially after manual circumcision. The incidence of infection after manual circumcision in the attending physician was higher than that in the chief physician.

## Conclusion

Circumcision, injection of anesthesia at the base of the penis, ligation of the penis, and postoperative wound infection may be the etiologies and triggers of secondary epidermoid cysts of the penis. Adults and phimosis patients may be high-risk groups. Lower-level surgeons may increase the odds of postoperative secondary epidermoid cysts of the penis, and it is recommended that surgery be performed by a clinically-experienced, higher-level surgeon. The indications for circumcision should be strictly evaluated and the operation should be performed as soon as possible, and the less invasive surgical method and anesthesia method should be selected. Reduce irrelevant operations during surgery and avoid wound infection after surgery.

## Data Availability

Almost all the data of this study has been included in the manuscript. Since the patients did not agree to disclose personal privacy information, this study cannot provide relevant personal privacy information including the patient's name, telephone number, home address, etc.
